# Comprehensive list of metabolites measured by DI-FTICR mass spectrometry in thyme plants with contrasting tolerance to drought

**DOI:** 10.1016/j.dib.2017.04.039

**Published:** 2017-04-29

**Authors:** Parviz Moradi, Brian Ford-lloyd, Jeremy Pritchard

**Affiliations:** a Research Division of Natural Resources, Zanjan Agricultural and Natural Resources Research and Education Centre, AREEO, Zanjan, Iran; b School of Biosciences, University of Birmingham, UK

**Keywords:** Metabolites, DI-FTICR, Mass spectrometry, Thyme, Plant, Drought

## Abstract

This article contains data related to the main research entitled “Metabolomic approach reveals the biochemical mechanisms underlying drought stress tolerance in Thyme” (Moradi et al., 2017) [1]. Two thyme populations with contrasting drought tolerance were subjected to long term water deficit. Leaf samples harvested at the end of stress period and bi-phasic extraction carried out to get polar and non-polar fractions. Extracted samples were analyzed through Direct Infusion FT-ICR mass spectrometry. Date files comprise of four separate tables for all the putatively identified metabolites and their intensities in watered and droughted plants. *P*-values beside each *m/z* values indicate significances of difference between peak intensities of stressed and control conditions.

**Specifications Table**

**Table:** 

Subject area	*Biology*
More specific subject area	*Metabolomics*
Type of data	*Table*
How data was acquired	*DI-FTICR mass spectroscopy*
Data format	*Analyzed*
Experimental factors	*Freeze-dried leaf samples of thyme plants were grown in watered and droughted conditions*
Experimental features	*Metabolites extracted using the methanol: chloroform: water protocol, then loaded in FT-ICR directly.*
Data source location	*University of Birmingham, Birmingham, UK*
Data accessibility	*Data is accessible at*http://dx.doi.org/10.17632/96wp2zp489.1

**Value of the data**

• DI FT-ICR with less than 1 ppm mass accuracy and measuring metabolites has been rapidly used in plant metabolomics [2]. This technique has two considerable advantages; namely high mass accuracy and high mass resolution [3].

• Since metabolites contributed in drought stress response could be common, though these metabolites in addition to their folding change and p-value would be useful for other researchers. In fact, to design an experiment on other plants, particularly targeted metabolomics experiments are certainly useful.
• These dataset have broad applications in identifications of transcriptomes and pathways involved in stress response mechanisms.

## 1 Data

Thyme leaf metabolites are presented in two classes of polar and non-polar metabolites. *M/z* value of metabolites ranged 70–590 *m/z* for polar fraction and 70–2000 *m/z* for non-polar fraction of extracted samples. Peak intensities in control (watered) and droughted grown plants are presented in the tables. *T*-test was used to identify the significant differences between the watered and droughted metabolites [1].

## 2 Experimental design, materials and methods

**Fig. S1:**
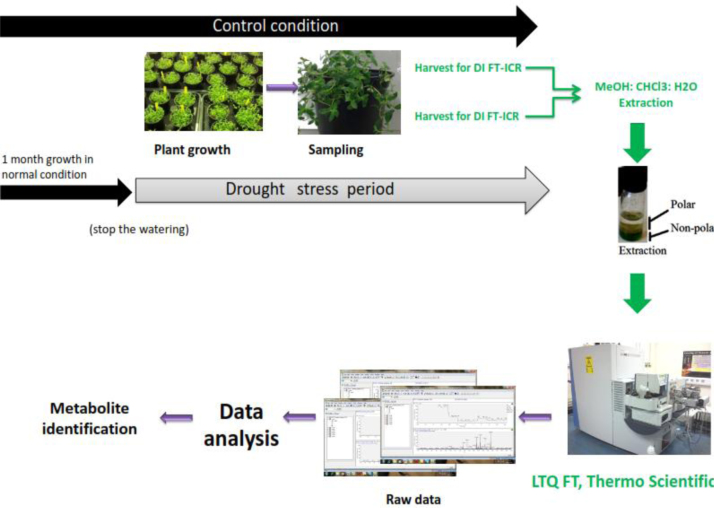
Metabolomics workflow used to acquire the dataset.

*Thymus vulgaris* and *Thymus serpyllum* (as representative of drought sensitive and drought tolerant plants respectively) were grown in a growth room with a 16:8 (light: dark) cycle and a temperature of 22 °C and watered with tap water weekly. Drought stress was applied as described previously [4]. Similar aged leaves of six individual plants as biological replicates were harvested for FTICR profiling at the end of drought stress period (Fig. S1).

Freeze dried samples were extracted using the methanol: chloroform: water protocol and separated extracts to polar and non-polar fractions. Polar extracts were dried with a vacuum concentrator (Thermo Savant, Holbrook, NY, USA) and non-polar extracts were dried under a stream of dried nitrogen gas. The dried extracts were stored at −70 °C until analysis.

Three technical replicates containing 10 µl aliquots from each polar and non-polar samples were analyzed using a hybrid 7-T Fourier Transformed Ion Cyclotron Resonance Mass Spectrometer (LTQ FT, Thermo Scientific, Bremen, Germany) equipped with a chip-based direct infusion nanoelectrospray ionisation assembly (Triversa, Advion Biosciences, Ithaca, NY). ChipSoft software (version 8.1.0, Advion Biosciences) was controlling the Nanoelectrospray conditions which had 200 nL/min flow rate, 0.3 psi backing pressure, and +1.7 kV electrospray voltage for positive ion analysis and −1.7 kV for negative ions.

Raw dataset were subjected to data analysis as follows:

### 2.1 Pre-processing

In order to process the mass spectra generated, 2 technical replicates out of 2 with an 80% sample filter were retained (peaks occurred at least 80% of samples within group independently). Next, raw data was subjected to custom-written code including sum of transient files and their process [5]. Then, processed transient data files were submitted to custom written codes in MATLAB (SIM-stitch algorithm version 2.8). Three more MATLAB scripts were applied to datasets, which referred to peak filtering [6]. At this stage, a peak list and a peak matrix were generated. The peak list comprised two columns, namely *m/z* (mass to charge) and related intensities. The peak matrix consisted of a multivariate dataset that recorded all the peaks detected for each biological replicate.

### 2.2 Metabolite identification

The peak mass list, along with peak intensities, were submitted to the Mi-Pack software package [7] to identify. For each given accurate mass within the peak list, the correct number of empirical formulae were calculated by implication of seven ‘golden rules’ [8]. It must be noted that, despite the high mass accuracy, one mass may linked to different elemental formula, or even similar formula but different structures. Hence, in this paper, for results tables, all the possible compounds have been inserted. For instance, for *m/z*=128.0108 all forms of alanine namely D-alanine, L-alanine and beta-alanine are considered and FTMS cannot distinguish between these isomers.

Complete list of metabolites for *T.vulgaris* as sensitive plant were presented in Tables S1 and S2 for polar and non-polar dataset respectively. Likewise, Tables S3 and S4 demonstrates the polar and non-polar metabolites for tolerant thyme (*T. serpyllum*) [9]. These data is available at http://dx.doi.org/10.17632/96wp2zp489.1.
