# Supplementary material for: Comprehensive list of metabolites measured by DI-FTICR mass spectrometry in thyme plants with contrasting tolerance to drought
Source: Data Brief. 2017 Apr 29;12:438–41. doi: 10.1016/j.dib.2017.04.039 (PMC5426011; doi:10.1016/j.dib.2017.04.039)
Supplement: Supplementary file 1 — Supplementary material [file mmc1.pdf]

CONFLICT OF INTEREST DECLARATION AND AUTHOR AGREEMENT FORM

It is important that you return this form upon submission. We will not publish your article without completion and return of this form.

Title of Paper: Comprehensive list of metabolites measured by DI-FTICR mass spectrometry in thyme plants with contrasting tolerance to drought

Please tick one of the following boxes:

☒ We have no conflict of interest to declare.

☐ We have a competing interest to declare (please fill in box below):

This statement is to certify that all Authors have seen and approved the manuscript being submitted. We warrant that the article is the Authors' original work. We warrant that the article has not received prior publication and is not under consideration for publication elsewhere. On behalf of all Co-Authors, the corresponding Author shall bear full responsibility for the submission.

This research has not been submitted for publication nor has it been published in whole or in part elsewhere. We attest to the fact that all Authors listed on the title page have contributed significantly to the work, have read the manuscript, attest to the validity and legitimacy of the data and its interpretation, and agree to its submission to the *Journal of Data in Brief.*

**Author Signature**	**Print Name**
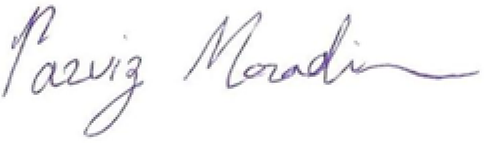	Parviz Moradi

☒ Please check this box if you are submitting this on behalf of all authors.
